# Computational analysis of the sequence-structure relation in SARS-CoV-2 spike protein using protein contact networks

**DOI:** 10.1038/s41598-023-30052-w

**Published:** 2023-02-17

**Authors:** Pietro Hiram Guzzi, Luisa di Paola, Barbara Puccio, Ugo Lomoio, Alessandro Giuliani, Pierangelo Veltri

**Affiliations:** 1 Department of Surgical and Medical Sciences, Magna Graecia University of Catanzaro, Catanzaro, Italy; 2 Unit of Chemical-Physics Fundamentals in Chemical Engineering, Department of Engineering, Universita Campus Bio-Medico di Roma, via Alvaro del Portillo 21, 00128 Rome, Italy; 3 Environment and Health Department, Istituto Superiore di Sanita, Rome, Italy; 4 Department of Computer, Modeling, Electronics and System Engineering, University of Calabria, Rende, Italy

**Keywords:** Computational models, Network topology, Protein structure predictions, Proteins

## Abstract

The structure of proteins impacts directly on the function they perform. Mutations in the primary sequence can provoke structural changes with consequent modification of functional properties. SARS-CoV-2 proteins have been extensively studied during the pandemic. This wide dataset, related to sequence and structure, has enabled joint sequence-structure analysis. In this work, we focus on the SARS-CoV-2 S (Spike) protein and the relations between sequence mutations and structure variations, in order to shed light on the structural changes stemming from the position of mutated amino acid residues in three different SARS-CoV-2 strains. We propose the use of protein contact network (PCN) formalism to: (i) obtain a global metric space and compare various molecular entities, (ii) give a structural explanation of the observed phenotype, and (iii) provide context dependent descriptors of single mutations. PCNs have been used to compare sequence and structure of the Alpha, Delta, and Omicron SARS-CoV-2 variants, and we found that omicron has a unique mutational pattern leading to different structural consequences from mutations of other strains. The non-random distribution of changes in network centrality along the chain has allowed to shed light on the structural (and functional) consequences of mutations.

## Introduction

Severe Acute Respiratory Syndrome Coronavirus 2 (SARS-CoV-2) has accounted for over 657 million infections and over 6.8 million deaths at the end of the 2022 (Data from https://covid19.who.int)^1^. SARS-CoV-2 is a large enveloped coronavirus (family-Coronaviridae, subfamily-Coronavirinae) with non-segmented, single-stranded, and positive-sense RNA genomes^2^. SARS-CoV-2 is composed of the spike (S), nucleocapsid (N), membrane (M), and envelope (E) proteins, of 16 non-structural proteins (NSP1-NSP16), and six accessory proteins (NS3, NS6, NS7a, NS7b, NS8, and ORF10). The Spike protein (S) infects human cells by binding the ACE2 human receptor^3–5^. During the pandemic, many mutant strains emerged, the vast majority of which had a very short life span and limited spatial distribution. In very few cases, the emerging mutant became the most common strain, with the sudden disappearance of the other variants.

Viruses, with their often small genomes and error-prone replication mechanisms, adapt very rapidly to changing micro-environmental cues. Moreover, the huge number of replications make it possible to observe their ‘natural evolution in real time’ as they acquire antiviral drug resistance or mediate persistent infection through escape from T and B cell immune system responses to infection^6^. This behaviour can be equated with a sort of Darwinian struggle for life in which ‘the fittest strain’ is the most efficient in terms of reproductive success. In the case of viruses, reproductive success is strictly related to its infective power, i.e. on the ability to enter into the host cells and reproduce. One consequence of this is the tendency of emerging infections to become milder in terms of their pathological effect (in some cases creating a symbiotic relationship^7,8^) thereby enabling more rapid diffusion across host (in this case human) populations. The above considerations allow to understand why proteins at the interface between viral particle and host cell are the key to understanding the evolution of the viruses.

The importance of S protein for infectivity and the consequent spread of the SARS-CoV-2 virus, and the fact that vaccines are tailored upon this protein, made S protein the privileged point of departure for the study of natural history of viruses in structural/bioinformatic terms. Sequence analyses of S protein have revealed the emergence of new SARS-CoV-2 mutation hotspots whose random or selection-driven character is hotly debated^1,9–11^. Here, we focus on a number of selected variants: Alpha, Beta, Gamma, Delta, and Omicron. These variants have different transmission rates, evolutionary patterns and levels of vaccine resistance and became predominant one after the other. Alpha prevailed over the original Wuhan strain, then Delta overcame Alpha and Omicron became virtually the only one left. The Omicron variant, identified in February 2022, has spread faster than earlier variants^12,13^ while its effects have proven to be less severe than previous strains. The Delta variant, isolated in a region of India in October 2020, has emerged as the dominant global variant alongside the Alpha^14^.

Since April 2021, the literature has been enriched by many works focusing on the impact of variants on SARS-CoV-2 S protein modification^15–19^ and the importance of mutations at the sequence level has been considered a crucial step in the analysis and prediction of variants. These studies were made possible thanks to the availability of a large volume data sets. The study of mutational landscape at the sequence level has been facilitated by the large volume of data stored in public databases (such as the GISAID database)^20^. On the contrary, the impact of the mutation at the structural level suffers from the lack of experimental data on protein structures. Existing structures stored in PDB database^21^ are mainly related to various structural domains of the Spike protein and its mutations^17^.

We present a systematic sequence-structure analysis of Spike protein for the three variants Alpha, Delta, and Omicron. We analysed the variants according to the following procedure: (i) we first checked the mutual differences between strains in sequence space; (ii) we computed the between strain differences in terms of residue contact network distances; (iii) we checked the existence of a general linear relation between sequence and structure metrics; (iv) we built up a phenomenological sequence/structure relation in terms of PCN descriptors of single mutated residues. We relied on our experience in Protein Contact Network (PCN) framework to perform the first sequence-structure analysis. Protein Contact Networks (PCNs)^22,23^ can catch the protein structure modularity at the basis of domain functional partition of protein molecular structures and allosteric regulation^24–26^. We compared the selected variants in terms of network invariants stemming from the PCN approach, complementing the classical sequence-based comparison. The distance-to-distance correlation analysis^27^ on global sequence and structure has 21 mutated residues (while the other two strains only 6 and 4 point mutations)hence an outlier pointing to a clear separation (in both sequence and structure metrics) of this strain with respect to the other two. Focusing on changes in the Eigenvector Centrality (EC) descriptor^28^, the unique mutational pattern of Omicron with its highly non-random distribution of EC changes emerged as a cue for rationalising the functional consequences of the observed mutations. We report a marked excess of centrality value in the Receptor Binding Domain (RBD), and a decrease in the cleavage-allosteric region, pointing to a significant restructuring consistent with phenotype changes (vaccine escape, lower lethality) and finally observed structural modifications of the Omicron strain^29^

### Related work

The study of protein sequence-structure (also referred to as Protein sequence-structure analysis - PSSA -) consists of an integrated analysis of protein sequences and structures. In recent years extensive research has been devoted to prediction by means of sequence comparison and alignments of sequences and structures^30–32^. The current outbreak of COVID-19 has opened up an unprecedented field of application for such methods. In^33^, the authors analysed Membrane (M) and Envelope (E) proteins of COVID-19 and the comparison to *homologous* proteins in MERS, SARS, and bat viruses, and found that many regions of E and M proteins of SARS-CoV-2 are similar to SARS and bat ones. Conversely, the MERS virus *proteins* differ in many respects.

Cherian et al.^34^, analysed four mutations of the Spike protein (L452R, T478K, E484Q and P681R), during the second wave of COVID-19 in Maharashtra (India). In particular, focusing on the impact of these mutations on the RBD domain structure, they found a possible increase of the ACE2 binding affinity in L452R, T478K, E484Q mutation, while postulating an increased transmission rate for P681R. The analysis of mutations of Spike protein has also been also treated in^35,36^, focusing on mutations affecting antigenicity. Ortuso et al.^17^ found certain Spike mutations in the RBD : S477N, N439K, N501Y, Y453F, E484K, K417N, S477I and G476S. Among these, they found that mutation N501Y, in particular, is one of the characteristic features of the SARS-CoV-2 Delta. Using mutation analysis for SaRS-CoV-2, Di Giacomo et al.^37^ reported a study on T478K mutation of S protein, integrating both sequence and structure analysis. All the above studies have employed a local (typical of structural biology and biochemistry studies) approach aimed at rationalising a structural (and/or functional) phenotype based on mutations present in the primary sequence.

The study of the mutations has been stimulated by the availability of a large amount of data over the world and each lineage has been characterised in terms of mutation, spatial distribution, and impact on disease evolution and transmissibility. For instance, the mutations of the Delta variants on S protein have shown a reduction in the reaction to antibodies^38^. Similarly, the mutation-based analysis for the Gamma variant evidenced a neutralising reduction of some antibodies^39^.

Moreover, the effects of the mutations on the vaccination have been investigated in many works such as^4,40–43^. Genomics sequence integration has been proposed in^44^ where a library of human antibodies and topological analysis of the sequences evidenced the evolution for S proteins and their impact on vaccination. They found that most common variants present the strengthening of infectivity and vaccine escape on the RBD domain of S protein. They also found that infectivity strengthening results from the evolution of the virus, and the emergence of possible vaccine-escape mutations is more likely to occur in highly vaccinated populations. Moreover, in^45^ the authors point to the ability of the Omicron strain to mutate in order to reduce the effect of the neutralising antibodies, while keeping a close affinity with the ACE2 receptor^45^.

In this work we used sequence-structure analysis and a systemic perspective. We some might argue that, since we consider only three mutant strains, we have little information^28^, making the overall sequence structure-relation severely biased. However, the computation of network centrality descriptors at the level of single mutated residues provides allow a direct appreciation of structural consequences of each sequence change. Indeed, we noted that the same point mutation presents many different behaviours in terms of topological changes of the network representing the S protein. This is a proof-of-concept of the unique feature of the proposed approach to translate purely local information into systemic terms.

## Results and discussion

### Sequence analysis

We analysed the sequence of the Spike protein considering three selected variants (Alpha, Delta, Omicron) in three different states (Closed, Open 1RBD-Up, Complex with ACE2). We obtained the best multiple alignment of considered sequences using Clustal Omega routine^46^. Afterwards, we created a distance matrix from a multiple sequence alignment, calculating the evolutionary distance between each pair of sequences in a multiple sequence alignment^47^.

### Structure analysis through protein contact networks

**Figure 1:**
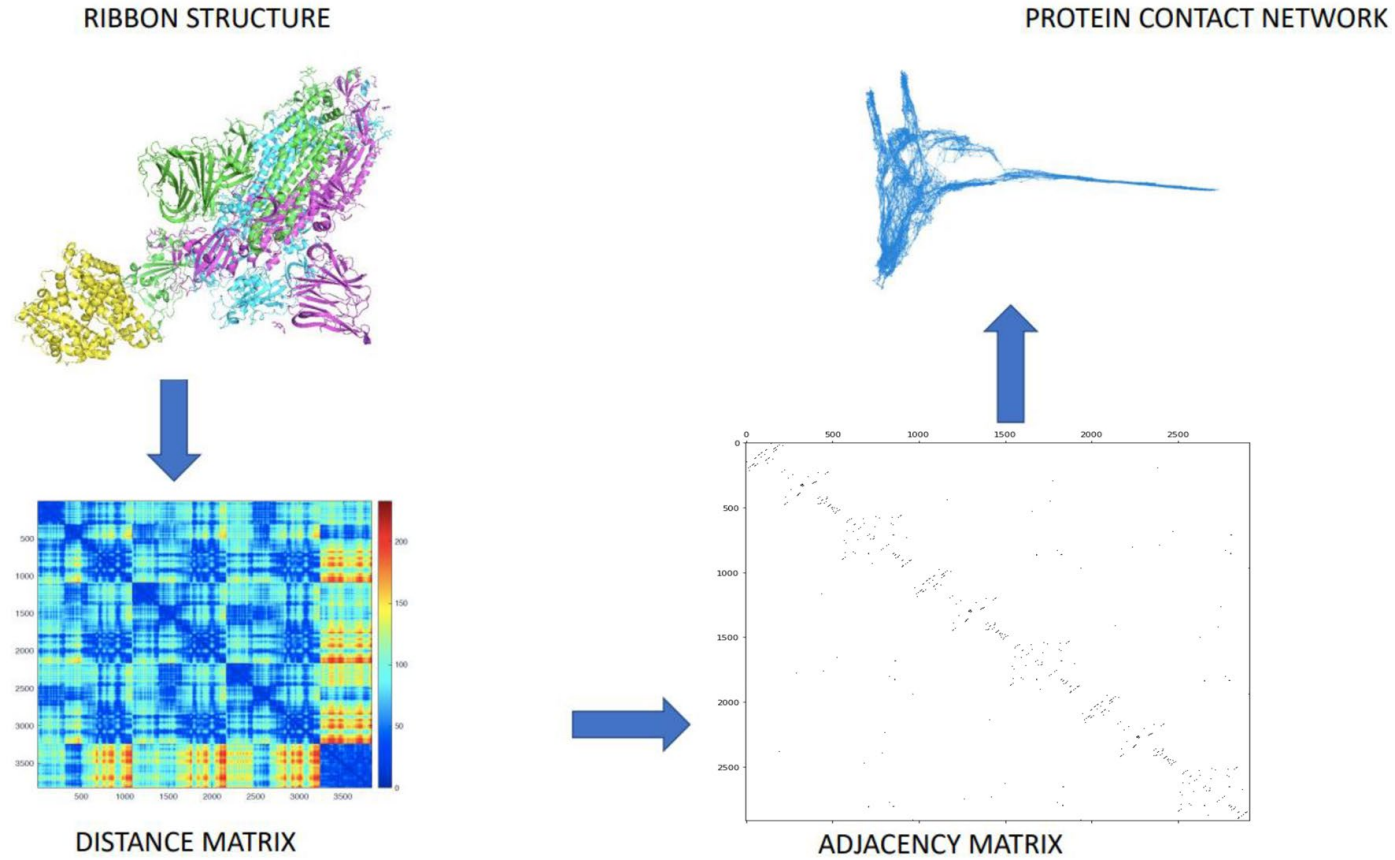
Scheme of the PCN construction on the close conformation of SARS-CoV 2 spike protein (PDB code 6vyb): starting from the structure (upper left). it is possible to compute the distance matrix (lower left), then the adjacency matrix (lower right) and finally the PCN (upper right).

We build PCNs using PCN-miner as depicted in Fig. 1. (PCNs)^22^ allow us to model a protein structure into a graph that can be analysed^22,23,48^. PCNs are networks whose nodes represent the $C-\alpha$ atoms of the backbone of proteins, while their edges represent a relative spatial distance between 4 and 8 angstroms. Topological descriptors of PCNs, such as centrality measures, are used to discover protein properties such as allosteric regions^24,48,49^. The structural distances between PCNs are computed by means of the Frobenius metric, which stems from the pair-wise comparison across 18 adjacency matrices (6 mutants and 3 aggregation states). The Frobenius metrics indicates the number of corresponding pairs of residues differing in the two structures. The Frobenius norm between two matrices is defined as the square root of the sum of the absolute squares of their elements.

#### Correlation between sequence and structure distance matrices

**Figure 2:**
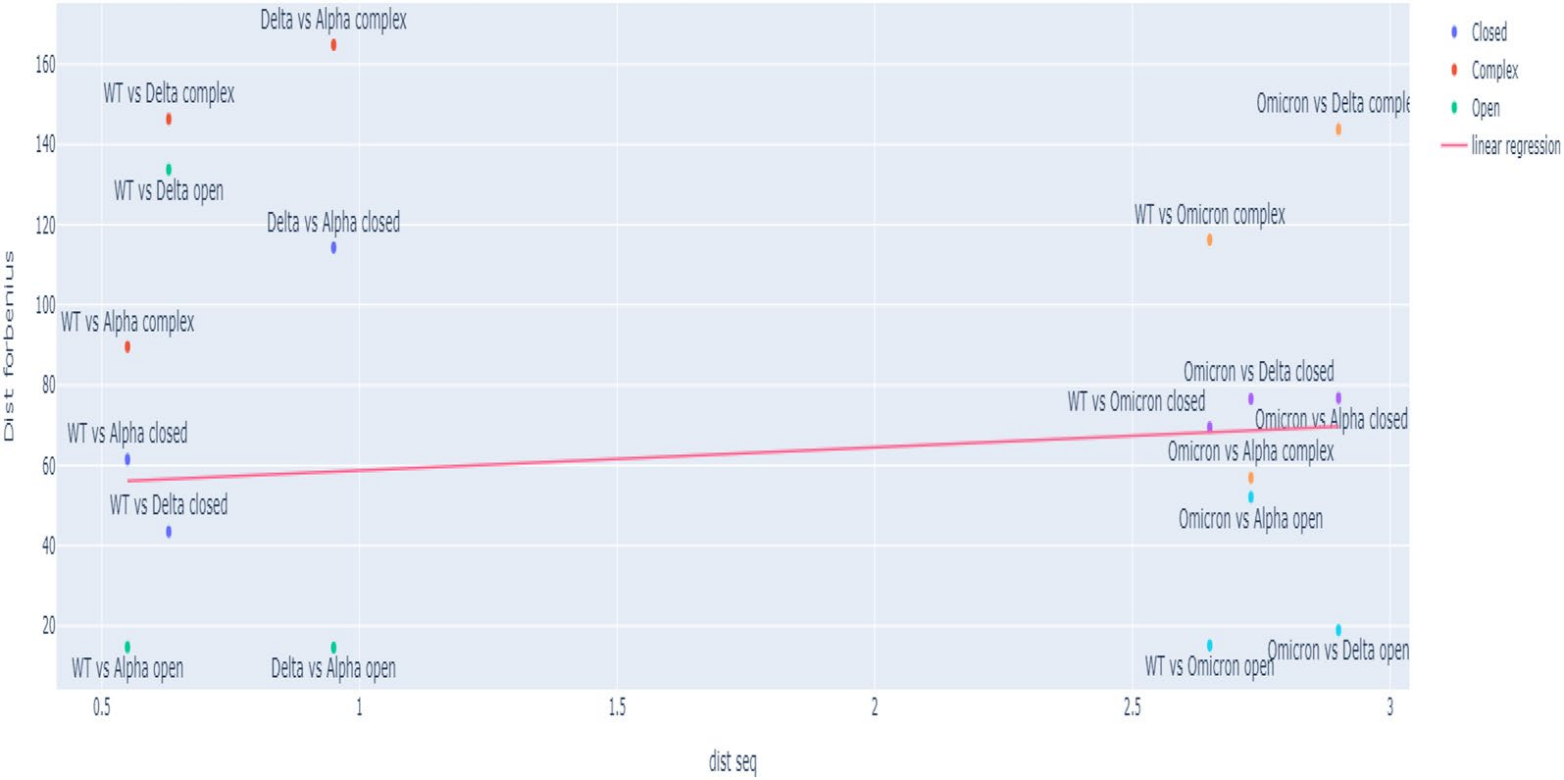
The x-axis of the Figure represents the distance of the sequences while the y-axis the distance of the structures. Each point represents the correlation of a pair sequence/structure. We report the wild type, alpha, delta and omicron variant and three structural conformations open, closed and complex.

We studied the correlation between the sequence distances and the related structure distances, as shown in Fig. 2. The x-axis of the Figure represents the pair-wise sequence distances that are identical for the three aggregation states and depend only upon the differences in the primary structure, while the y-axis corresponds to the pair-wise mutual distances relative to the three aggregation states for the mutants. Each point represents the correlation of a pair sequence-structure. We report results for the wild-type, alpha, delta and omicron variants and the three different structural conformations, open, closed and complex with ACE2.

The Figure shows that there is no correlation between sequence and structure. In other words, the entity of sequence distance has no explanatory content for the resulting structural distance. We should note that the Omicron variant is by far the most mutated species of all other forms bearing 21 mutated locations, while alpha and delta strains have 6 and 4 mutated locations, respectively. The range restriction effect completely hides any general sequence/structure relation that collapses to the pure registration of the singular position of the Omicron strain in both sequence and structure spaces. This well-known fact^50^ evidence of the continuity breaking of sequence distance with the oncoming of a mutant (Omicron) bearing a higher number of mutations than the others is a puzzling event pointing to strong selective pressure regarding random mutational events. The Omicron strain is notably different from the others, as unfortunately shown by the poor performance of the vaccines^51,52^. Second, we note that the wider distances of structures (y-axis) are related to the complex of S and ACE2 proteins; this may be related to the higher infectivity rate of this strain. This result tells us that structural (and consequently functional) differences are highly context-dependent at the fine scale of analysis, preventing any simple extrapolation from sequences.

#### Network invariants

Having assessed the singular position of Omicron vis-à-vis Alpha and Delta strains by both sequence and structure (entire network wiring) spaces, network invariant analysis enables us to explain the structural changes. The change in network invariants regarding the initial Wuhan strain was estimated in terms of log ratio. Thus a value of zero (0 in number) corresponds to no change. Positive and negative values point to an increase or decrease, respectively. We calculated main centrality measures for each node: Closeness (CC), Eigenvector EC, and Betweenness (BC) Centrality^53,54^. The Closeness Centrality (CC) measures how close the nodes are to each other in term of shortest paths. The Eigenvector Centrality (EC) indicates the importance of a node in a network. The Betweenness Centrality (B)C estimates show how many shortest paths go through a given node, thereby revealing its important role in signal transmission throughout the network. Recently, Barozi and coworkers applied these centrality metrics to analyse the Molecular Dynamics of the Omicron S-protein by identifying a specific evolutionary pattern towards an increased allosteric regulation of the S-protein RBD-hACE2 binding^55^.

**Figure 3:**
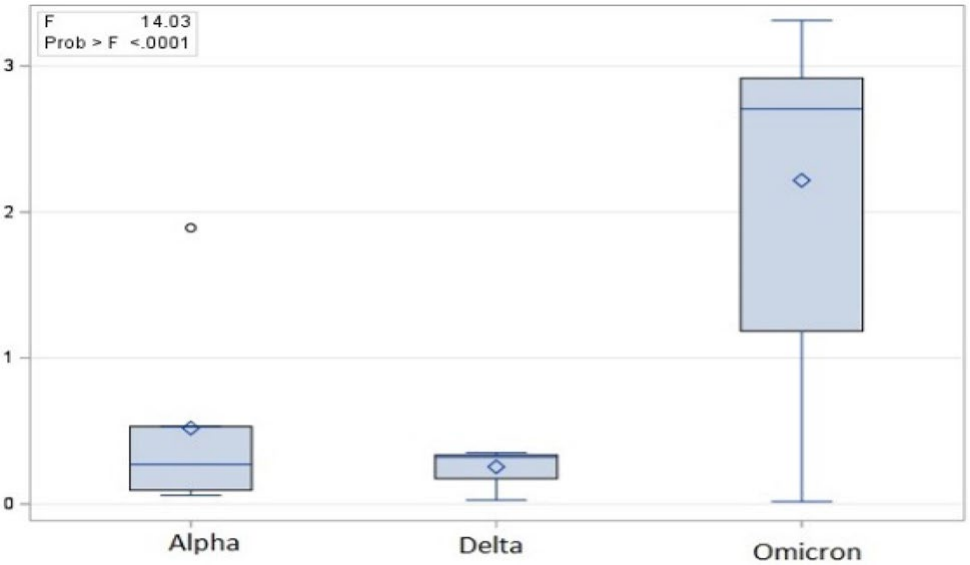
The absolute values of eigenvector centrality (EC) variations relative to the three analysed strains are reported as box-plots. In terms of the changes it is evident that the position of Omicron is quite different from that of the other strains. The use of module values is crucial for a statistically significant result (F = 14.03, $p<$ 0.0001).

Only the EC showed a striking variation from a global null effect with Omicron, highlighting a marked and statistically significant difference regarding the other strains ($F = 14.03$, $p < 0.0001$ as for an absolute change in EC). It is worth stressing that this result has no necessary relation with the number of mutated sites, given that it corresponds to the average change per mutated residue (see Table 1, and Fig. 3).

We also report in Table 2 that the EC changes, setting a threshold of relevant change at EC = |2| and considering values less than $-2$ (i.e., < − 2) as ‘negative’ and greater than 2 (i.e. values > 2) as ‘positive’, we note that only Omicron mutations lead to the changes in their role in PCN wiring. The distribution of these mutations is far from random; positive changes are only present in RBD domain, while negative changes concentrate on the splicing domain^29^. The Eigenvector Centrality (EC) of a node can be equated to the loading of a variable on the first principal component of a multivariate data set^56^. Thus, the increase of EC of a set of nodes of the same protein domain corresponds to a drastic contraction of the domain marked by an increase in the amount of structural variance explained by the first component. On the contrary, a *proxy* of structural relaxation corresponds to a decreased value of EC coordinates.

This implies that the Omicron RBD has a more compact structure than the original strain, which is consistent with observations in other works, such as^29^. The analysis is labor intensive as well as dependent on the availability of the structures from the PDB database. In addition, we observed relaxation of the splicing domain. Considering that the splicing domain encompasses the allosteric site of spike protein, we achieve a highly consistent structural explanation of this peculiar phenotype of Omicron. We also observe that Omicron presents these characteristics: (i) from a molecular point of view it exhibits stabilisation of RBD and increased sensitivity to microenvironment allosteric signalling (relaxation of splicing domain); (ii) from a phenotypic point of view, data from the literature reports higher infectivity and vaccine escape. Thus, we hypotesise that molecular changes determine the changes to the phenotype.

A final remark is related to the considerably different EC change provoked by the mutation of the same residues by the same amino acid substitution as for 501 and 614 positions in the three strains (Table 2). While these mutations do not provoke any notable EC change in Alpha and Delta strains, they culminate in a drastic EC change in Omicron. This provides evidence of the ‘context dependent’ character of network invariants of mutation patterns with respect to the purely local consideration of both the sequence-based and global structural views.

## Methods

### Protein contact networks

A protein structure can be represented as a complex three-dimensional object formally defined by the coordinates in the 3D space of its atoms. Despite the wide availability of data on protein molecular structures, the protein structure-function relationship is far from fully understood. For this reason, it is necessary to define simple descriptors that can describe protein structures with few numerical variables. Structure and function are based on the complex network of inter-residue interactions, where residues are identified by amino acid sequences^22^. The interaction of residues, therefore, is the way to define protein contact networks (PCN) that represent the protein structure by means of $\alpha$-carbon location. The spatial position of $C_{\alpha }$ is still reminiscent of the protein backbone, and this allows us to also highlight the most important features of the three-dimensional structure. Starting from spatial distribution of the $C_{\alpha }$, a distance matrix *d* is evaluated where each $d_{i,j}$ represents the Euclidean distance in the 3D space between the *i*-th and *j*-th residues, defined as

1 $$\begin{aligned} d_{i,j}= \sqrt{((x_i-x_j)^2)+((y_i-y_j)^2)+(z_i-z_j)^2)} \end{aligned}$$

where $(x_i,y_i,z_i)$ and $(x_j,y_j,z_j)$ respectively are the Cartesian coordinates of residue *i* and *j*. Matrix *d* is used to define a Protein Contact Network^22^^57^. It is possible to build up adjacency matrix A, whose generic element is defined as:

2 $$\begin{aligned} A_{ij} = \left\{ \begin{array}{rl} 1 &{} \quad \text{ if } \quad 4 \, \le d_{ij} \le \, 8 \\ 0 &{} \quad \text{ otherwise } \end{array} \right. \end{aligned}$$

Thus, we define a link between two residues *i* and *j* if their mutual distance lies between 4 and 8 Å. The lower end excludes all covalent bonds, which are not sensitive to environmental change (hence to protein functionality), while the upper end eliminates of weaker non-covalent bonds (hence not significant for protein functionality).

The adjacency matrix of a graph is unique regarding the ordering of nodes. With proteins where the order of nodes (residues) corresponds to the residue sequence (primary structure), the evidence shows that its corresponding network is unique: this establishes a one-to-one correspondence between the protein and its network.

In the case of SARS CoV-2 spike protein, this formalism has been used to detect the allosteric site of the S protein^58^ through an integrated structural/dynamic approach^59^.

Finally, we consider the distance between two PCN as the Frobenius distance of their adjacency matrices.

### Datasets

We consider SARS-CoV-2 genomic sequences extracted from GISAID^20^ database on March 2022. Sequences used in this work can be found at https://github.com/hguzzi/Multiscalemodelling. We downloaded thirteen protein structures from the Protein Data Bank (PDB https://www.rcsb.org/): 6vxx, 7wk2, 7sbk, 7fet, 6vyb, 7edf, 7w92, 7tgw, 7df4, 7fem, 7wk4, 7w98, 7vxm. Coordinates of the Carbon-$\alpha$ atoms were used to obtain PCNs.

### Sequence comparison

Sequence alignment was performed by the Smith-Waterman and the Clustal Omega algorithm^46^. Regarding pair-wise alignment, we used EMBOSS^47^ (European Molecular Biology Open Software Suite), that is a high-quality package of open source software tools for molecular biology. It uses the Smith-Waterman algorithm (changed for speed enhancements) to calculate the local alignment of two sequences. For multiple alignment, we used Clustal Omega, a multiple sequence program that uses seeded guide trees and HMM profile-profile techniques to generate alignments between three or more sequences. The pair-wise alignment tool contains a file that reports input parameters (i.e., two sequences in FASTA format), alignment obtained and expressed as a percentage. PCN-Miner software http://github.com/hguzzi/PCN-MINER was used to build PCNs^26^.

### Centrality measures

We considered following centrality measures with respect to PCNs: degree, closeness, betweenness and eigenvector.

Degree centrality is the number of adjacent nodes to $w_i$ which can be defined as follows.

$$\begin{aligned} C_{deg}(w_i)=deg(w_i). \end{aligned}$$

Closeness centrality is to be considered as a central node close to the others in terms of distance. Formally, the closeness centrality of node $w_i$ is the reciprocal of the average shortest distance to $w_i$ over all $n-1$ reachable nodes, i.e.

$$\begin{aligned} C_{closeness}(w_i) = \frac{n-1}{\sum _{j=1}^{j=n-1,j \ne i}d(w_i,w_j)}. \end{aligned}$$

where $d(w_i,w_j)$ is the shortest distance between $w_i$ and $w_j$.

Given an adjacency matrix $\mathcal {A}$, the relative centrality score a node *v* can be defined as:

$$\begin{aligned} x_{v} = \frac{1}{\lambda }\sum _{w \in Neigh(v)} x_w = \frac{1}{\lambda }\sum _{w \in \mathcal {G}} \mathcal {A}_{v,w} x_w \end{aligned}$$

Eigenvector centrality estimates the influence of a node in a network. It scores the nodes of a network on the basis of the idea that high-scoring nodes contribute more to the score of the node than connections to low-scoring nodes. It has been shown that eigenvector centrality identifies the role of residues in allosteric signal transmission, both on a local and global scale^60^.

Given an unweighted undirected graph *G* and its adjacency matrix $\mathcal {A}$ we can estimate the EC ($x_{v}$) for each node *v* as

$$\begin{aligned} x_{v} = \frac{1}{\lambda }\sum _{w \in Neigh(v)} x_w = \frac{1}{\lambda }\sum _{w \in \mathcal {G}} \mathcal {A}_{v,w} x_w \end{aligned}$$

where *Neigh*(*v*) is the set of neighbours of *v*, and $\lambda$ is a constant. The previous equation may be written as in vector notation as the eigenvector equation $\textbf{Ax} =\lambda \textbf{x}$, where $\lambda$ is an eingenvalue for which a non-zero eigenvector solution exists.

Betweenness centrality is defined as follows:

$$\begin{aligned} C_{betweennes}(w_i) = \sum _{i \ne j \ne k} \frac{ \sigma _{j,k}(i)}{\sigma _{j,k}} \end{aligned}$$

where $\sigma _{j,k}$ is the total number of the shortest paths from node $w_j$ to node $w_k$ and $\sigma _{j,k}(i)$ is the number of those paths that pass through *i*.

## Conclusion

It is widely recognised that mutations of protein sequences impact first on their structure and then their function. The recent pandemic has provided an unprecedented scenario for the analysis of protein mutation, focusing on the mutations of SARS-CoV-2 viral proteins. We relied on this information to give a proof-of-concept of the ‘quantum-leap’ in terms of extraction of hypothesis on structure-function relations provided by a mesoscopic approach, such as PCN. Our results clearly show that mutations in the Omicron sequence cause the increase and the decrease of EC in two distinct regions. Moreover, as evidenced by the ANOVA test, Omicron mutations, regardless of the number and region, cause a more marked shift in EC, confirming their different pattern of mutation. As regard sequence/structure/function protein studies, this result leads to a shift from episodic local considerations of single mutations to a context-dependent evaluation of structural consequences of point mutations along the lines of Quantitative Structure Activity (QSAR) studies of small organic molecules.

**Table 1:** The table reports mean and standard deviation for both real and absolute (|*EC*|) values of eigenvector centrality for the three strains.

Strain	Mean (EC)	Mean (|EC|)	SD (EC)	SD (|EC|)
Alpha	− 0.469	0.521	0.739	0.696
Delta	0.066	0.256	0.324	0.153
Omicron	0.988	2.147	2.201	1.015

It is worth noting the neat departure from no effect for the Omicron strain together with the elevated standard deviation (SD (EC)) of current values pointing to the presence of both highly positive and negative EC changes for mutated residues. On the contrary, for the other two strains mutations do not provoke any important change in EC.

**Table 2:** The EC change (logratio) with respect to the original strain for each mutated amino acid residue is reported.

Strain	Mutated residue	modEC	effect
Alpha	501	− 1.89267	Null
Alpha	570	− 0.16767	Null
Alpha	614	− 0.375	Null
Alpha	716	0.060667	Null
Alpha	1118	0.095333	Null
Delta	142	− 0.352	Null
Delta	452	0.321667	Null
Delta	614	0.321667	Null
Delta	950	0.028	Null
Omicron	67	− 0.786	Null
Omicron	142	− 1.08433	Null
Omicron	339	2.449667	Positive
Omicron	371	3.125667	Positive
Omicron	373	2.703333	Positive
Omicron	375	2.721333	Positive
Omicron	417	3.207	Positive
Omicron	440	2.709667	Positive
Omicron	493	2.906333	Positive
Omicron	496	2.876	Positive
Omicron	498	2.930667	Positive
Omicron	501	2.826	Positive
Omicron	547	1.134667	Null
Omicron	614	− 2.14667	Negative
Omicron	655	− 2.32667	Negative
Omicron	764	− 0.82067	Null
Micron	796	− 3.03567	Negative
Omicron	856	1.23533	Null
Omicron	954	0.017667	Null
Omicron	969	3.31266	Null

The absence of any relevant change in EC of the Alpha and Delta strain is worth than nothing, while 14/21 (67%) Omicron mutations imply a significant change with respect to the original strain. Moreover the distribution of positive and negative changes is far from random, positive changes being concentrated in RBD domain, while negative changes are only found in the splicing domain.

## Data Availability

The website https://github.com/hguzzi/Multiscalemodelling contains data and code used in this work. More material may be shared upon reasonable request. Please contact Pietro Hiram Guzzi hguzzi@unicz.it for any request.
